# Reproducibility and Applicability of Traditional Strength Training Prescription Recommendations

**DOI:** 10.3390/biology11060851

**Published:** 2022-06-02

**Authors:** Juan Ramón Heredia-Elvar, Juan Hernández-Lougedo, Luis Maicas-Pérez, Raúl Notario-Alonso, Manuel Vicente Garnacho-Castaño, Pablo García-Fernández, José Luis Maté-Muñoz

**Affiliations:** 1 Department of Physical Activity and Sports Science, Alfonso X El Sabio University, 28691 Madrid, Spain; jelvaher@uax.es (J.R.H.-E.); jhernlou@uax.es (J.H.-L.); lmaicper@uax.es (L.M.-P.); rnotario@uax.es (R.N.-A.); 2 Campus Docent Sant Joan de Déu, University of Barcelona, 08034 Barcelona, Spain; manuelvicente.garnacho@sjd.edu.es; 3 Department of Radiology, Rehabilitation and Physiotherapy, Complutense University of Madrid, 28040 Madrid, Spain; jmate03@ucm.es; 4 IdISSC—Instituto de Investigación Sanitaria del Hospital Clínico San Carlos, 28040 Madrid, Spain

**Keywords:** strength, sport performance, human performance, velocity, external load, internal load, training, repetitions, fatigue

## Abstract

**Simple Summary:**

One of the main problems that exercise professionals face is controlling and quantifying the real load of resistance training in an objective manner. Several authors have made recommendations aimed at improving strength for different populations. However, it is necessary to verify whether these recommendations can be implemented, completing them in their entirety. Therefore, the aim of this study was to verify the reproducibility of a resistance training protocol in the bench press exercise, based on traditional recommendations, analysing the effect of the muscle fatigue of each set and of the whole exercise protocol. Thirty participants performed a bench press exercise protocol of three sets with the maximum number of repetitions possible to muscle failure (with 2 min rests between sets), using a relative load corresponding to 70% 1RM determined through the mean propulsive velocity obtained from the individual load–velocity relationship. The conclusions of the study were that it was not possible to complete the same number of repetitions in each set for the same absolute load. Moreover, the level of fatigue generated through each set and its relationship with the capacity to recover in the established time could be different in each individual, showing an important coefficient of variation in each of the sets.

**Abstract:**

Background: The aim of this study was to verify the reproducibility of a resistance training protocol in the bench press (BP) exercise, based on traditional recommendations, analysing the effect of the muscle fatigue of each set and of the whole exercise protocol. Methods: In this cross-sectional study, thirty male physical education students were divided into three groups according to their relative strength ratio (RSR), and they performed a 1RM BP test (T1). In the second session (T2), which was one week after T1, the participants performed a BP exercise protocol of three sets with the maximum number of repetitions (MNR) possible to muscle failure, using a relative load corresponding to 70% 1RM determined through the mean propulsive velocity (MPV) obtained from the individual load–velocity relationship, with 2 min rests between sets. Two weeks later, a third session (T3) identical to the second session (T2) was performed. The MPV of each repetition of each set and the blood lactate level after each set were calculated, and mechanical fatigue was quantified through the velocity loss percentage of the set (% loss MPV) and in a pre-post exercise test with an individual load that could be lifted at ~1 m·s^−1^ of MPV. Results: The number of repetitions performed in each set was significantly different (MNR for the total group of participants: set 1 = 12.50 ± 2.19 repetitions, set 2 = 6.06 ± 1.98 repetitions and set 3 = 4.20 ± 1.99 repetitions), showing high variation coefficients in each of the sets and between groups according to RSR. There were significant differences also in MPVrep Best (set 1 = 0.62 ± 0.10 m·s^−1^, set 2 = 0.42 ± 0.07 m·s^−1^, set 3 = 0.36 ± 0.10 m·s^−1^), which significantly reduced the % loss MPV of all sets (set 1 = 77.4%, set 2 = 64%, set 3 = 54.2%). The lactate levels increased significantly (*p* < 0.05) (set 1 = 4.9 mmo·L^−1^, set 2 = 6 mmo·L^−1^, set 3 = 6.5 mmo·L^−1^), and MPV loss at 1 m·s^−1^ after performing the three sets was 36% in T2 and 34% in T3, with acceptable intrasubject variability (MPV at 1 m·s^−1^ pre-exercise: SEM ≤ 0.09 m·s^−1^, CV = 9.8%; MPV at 1 m·s^−1^ post-exercise: SEM ≤ 0.07 m·s^−1^, CV = 11.7%). Conclusions: These exercise propositions are difficult to reproduce and apply. Moreover, the number of repetitions performed in each set was significantly different, which makes it difficult to define and control the intensity of the exercise. Lastly, the fatigue generated in each set could have an individual response depending on the capacity of each subject to recover from the preceding maximum effort.

## 1. Introduction

One of the main problems that exercise professionals face is controlling and quantifying the real load of resistance training in an objective manner [1]. Different studies have described specific criteria regarding the prescription of resistance training [2,3]. Some of these criteria would be related to certain values of intensity, volume and recovery between sets and exercises, depending on the training level of the subjects, the goals established, etc.

In this regard, several authors have made recommendations aimed at improving strength for different populations, such as healthy adults [4,5] and people with obesity and type II diabetes [6,7,8]. These recommendations establish relative intensities equivalent to 60–70% of maximum effort (1RM), with 1–3 sets of 8–12 repetitions each (A Grade recommendation based on the evidence), setting a rest interval of 2–3 min between sets (C Grade recommendation based on the evidence) [9]. Two issues derive from these recommendations: (a) in some cases, the number of repetitions proposed is maximal, and, in other cases, the load allows completing the number of repetitions proposed inducing fatigue (the degree of fatigue is not defined), without reaching “exhaustion”; (b) in most cases, there is no specification of the modification of the absolute load with which each set of repetitions is performed, thus it would remain stable and invariable throughout each set [10,11].

Using a value of the relative intensity established by the percentage of the maximum effort (1RM) or by the maximum number of repetitions (MNR) per set implies a margin of error that would not allow obtaining accurate data of the relative intensity [12]. Therefore, it is necessary to determine whether the number of repetitions performed in the different sets (1–3), as a function of the proposed relative intensity, and with the recommended rest time, fits a degree of effort in which the subject reaches considerable fatigue, even exhaustion. That is, it is necessary to verify whether these recommendations can be implemented, completing them in their entirety.

In most of these recommendations, as well as in numerous studies, a similar maximum number of repetitions is indicated in all series without detailing the modification of the absolute load [10,11]. Other studies have reported a “modification of the load”, which was applied to complete a range of maximum repetitions, either throughout the sets or, in some cases, throughout the sessions, without specific data regarding the real load performed by each subject or the corresponding mean values of the groups [13,14].

Therefore, with the aim of controlling and quantifying the resistance training load in a more objective manner, the aims of the present study were to: (1) verify the reproducibility of a resistance training protocol in a pushing action for the upper limbs [bench press (BP) exercise], following traditional recommendations [5,9]; (2) analyse the effect of the muscle fatigue of each set and of the whole protocol; and (3) determine the intrasubject–intersubject variability in the different variables in the training protocol.

## 2. Materials and Methods

### 2.1. Study Design

**Figure 1:**
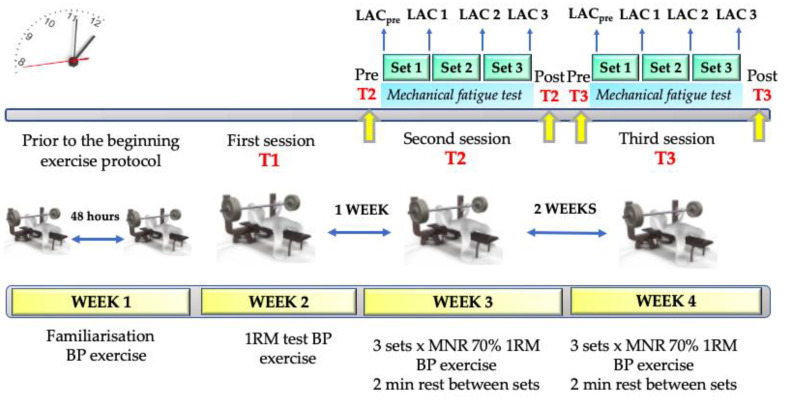
Study design. BP = bench press; LAC = blood lactate concentrations; 1RM = one-maximum repetition test; MNR = Maximum Number of Repetitions; Min = minutes.

The experimental design of this study consisted of the execution of three BP exercise protocols. In the first session (T1), the participants performed a test of progressive loads in BP to the 1RM of each subject, with the aim of determining the load–velocity relationship of each subject. In the second session (T2), they conducted a BP exercise protocol that consisted of the execution of three sets, in which they performed the MNR possible to muscle failure. Lastly, a third session (T3) identical to T2 was carried out, in order to explore the intrasubject variability of the exercise protocol. The three sessions were conducted on the same day of the week with a time interval of no more than two hours (±2 h) to evaluate the effects of the circadian rhythms [15]. The week prior to the beginning of the exercise protocol, two familiarisation sessions were carried out to allow the participants to familiarise with the BP exercise, with a separation of 48 h (Figure 1). The tests were conducted in the exercise physiology laboratory of the university, with a temperature of 18–22 °C and 40–55% humidity. The participants were initially briefed. One week was given for voluntary registration; the following week they performed the familiarisation, and then started the three tests, one per week.

### 2.2. Participants

Thirty healthy men (age: 22.56 ± 3.44 years; body mass: 76.61 ± 10.93 kg; height: 1.79 ± 0.06 m; BMI: 23.95 ± 2.73 kg·m^2^; and relative strength ratio (RSR) (obtained from 1RM strength/body mass): 1.06 ± 0.27), participated in the study. The subjects were divided into three groups based on their relative strength ratio (RSR): (1) High RSR group (*n* = 8) (RSR > 1.25); (2) Medium RSR group (*n* = 10) (1.25 > RSR > 0.95); and (3) Low RSR group (*n* = 12) (0.95 < RSR). All participants were students of the degree of sport sciences, and none of them had orthopaedic, metabolic or cardiorespiratory limitations that could limit their performance in the study. All of them knew how to technically perform a correct execution of the BP exercise. None of them used pharmaceutical drugs, food supplements or stimulating drinks during the study, and they were asked to restrain from eating two hours before the tests, only allowing them to drink water. Moreover, the day before each test, all subjects were requested to rest and to avoid any physical activity. In a previous session at the beginning of the study, all participants were informed about the tests to be performed, and they voluntarily signed the informed consent. The protocol of the study was authorised by the ethics committee of the university, following the principles of the Declaration of Helsinki [16].

### 2.3. Procedures

#### 2.3.1. One-Repetition Maximum (1RM) Test

Before conducting the 1RM test, there were two familiarisation sessions, in which one of the researchers explained how the BP exercise had to be performed. The technical execution of the BP exercise was as follows: in the supine position on the bench, with the hips and knees flexed and the feet on the bench; the arms slightly more open than shoulder width; the bar was brought down gently and slowly to the chest, right above the intermamillary line; the bar was held on the chest for approximately 2 s, in order to remove the bouncing effect and improve the reliability of the measurements [17]. The order of executing the concentric phase was verbally given by one of the researchers, counting the 2 s stop between the eccentric phase and the concentric phase. The participants were encouraged to perform the concentric phase at the maximum possible velocity, without lifting their shoulders or trunk from the bench and without bouncing. Before carrying out the 1RM test, the participants conducted a warm-up consisting of 5 min of low-intensity running and 5 min of joint mobility and dynamic stretching exercises, followed by one set of 10 repetitions of BP with a fixed load of 10 kg, and one set of 5 repetitions of BP with a fixed load of 20 kg. After the warm-up, a progressive load test in the BP exercise to 1RM (1RM test) was performed, obtaining the load–velocity relationship individually. A detailed description of the BP test protocol has been recently provided elsewhere [18].

#### 2.3.2. The 3 × Maximum Number of Repetitions (MNR) Exercise Protocol

The second test (T2) was performed one week later, and it consisted of performing three sets in the BP exercise against 70% 1RM; this load was determined through the MPV obtained from the individual load–velocity relationship. The repetitions to be carried out in each of the three sets were the maximum number of repetitions possible to muscle failure (MNR). The rest time between sets was 2 min. Exactly two weeks after T2, the third test was conducted (T3), repeating the test of maximum number of repetitions to muscle failure. The technical execution of BP in T2 and T3 was identical to that of the 1RM test.

#### 2.3.3. Blood Lactate Concentrations

The capillary blood samples (5 μL) to determine the concentrations of the lactate of each participant were extracted from the tip of the index finger before the warm-up and after each set of MNR test both in T2 and T3.

#### 2.3.4. Mechanical Fatigue Test

To quantify the mechanical fatigue induced by the T2 and T3 protocols, we used the percent change between pre–post exercise with an individual load that could be lifted at ~1 m·s^−1^ of the MPV (MPV at 1 m·s^−1^ Tests) in a BP. To obtain the individual load at 1 m·s^−1^, weights were lifted until this velocity was reached. We started with the barbell (10 kg) and increased the weight by 1.25–5 kg, performing 3 repetitions with each load, resting 3 min between loads. Sánchez Medina and González-Badillo (2011) [19] used the value of 1 m·s^−1^ based on the fact that it is a sufficiently high velocity, which is attained against medium loads (45–50% RM in BP), and it allows obtaining a good expression of the effect of loading on velocity; moreover, it is a relatively easy-to-move and well-tolerated load. The average MPV of the three repetitions before the exercise was compared with the average MPV of the three repetitions after the exercise [19]. All repetitions were performed at maximum velocity. The MPV at 1 m·s^−1^ BP Tests was measured (bar velocity values during the propulsive phase, defined as the portion of the concentric phase during which bar acceleration is ≥9.81 m·s^−2^).

### 2.4. Measurement Equipment

A previously validated and calibrated portable analyser was used to obtain the blood lactate samples (Lactate Pro 2 LT-1710, Arkray Factory Inc., KDK Corporation, Siga, Japan) [20,21].

The different BP tests were measured using a Smith device with a guided bar and multipower system (Matrix, Chácara Alvorada, Brazil), employing plates of 20, 10, 5, 2.5 and 1.25 kg (Matrix). Thus, the two ends of the bar were fixed, allowing only the vertical movement of the bar. To estimate the execution velocity of each repetition in the different tests, a previously validated optoelectronic instrument [22] was used, with a sampling frequency of 500 Hz (Velowin v.1.7.232, Instrumentos y Tecnología Deportiva; Murcia, Spain). The optoelectronic instrument was calibrated following the manufacturer’s instructions.

### 2.5. Variables Analysed

For T1, T2 and T3, the MPV was calculated for each repetition of each set (bar velocity values of the propulsive phase, defined as the portion of the concentric phase during which bar acceleration is ≥9.81 m·s^−2^). In addition, the following parameters were measured in T2 and T3: (1) blood lactate concentration before the test and after each set; (2) the number of repetitions per set; (3) MPV of the best (fastest) repetition of the set (MPVrep Best); (4) MPV attained at the last repetition of the set (MPVrep Last); and (5) loss of MPV (% loss MPV Set), defined as: (MPVrep Last–MPVrep Best)/MPVrep Best × 100.

### 2.6. Statistical Analysis

Second-order polynomials were used to establish the load–velocity relationship for each subject in the progressive load test to 1RM (T1). Then, the Shapiro–Wilk test was initially used to verify the normality of the variables. To analyse the different variables of the BP protocol (T2) in the whole group of participants, a single-factor repeated measures ANOVA was performed, comparing it with Mauchley’s sphericity test. For those cases in which the sphericity hypothesis was rejected, the univariate F-test was used, adjusting it with the Greenhouse–Geisser correction index. When significant differences were obtained between measurements, Bonferroni’s post hoc test was applied.

To analyse the T2 exercise protocol as a function of the different strength levels, a two-factor repeated measures ANOVA was performed for the time factor, applying Levenne’s test to assess the homogeneity of variances. Therefore, it was considered that there was an intersubject factor with 3 groups per level (High RSR, Medium RSR, Low RSR) and an intrasubject factor with the variable “sets” in 3 levels (set 1, set 2, set 3) (3 groups × 3 sets), also observing the effect of the interaction and applying Bonferroni’s post hoc test for pairwise comparison. Moreover, the effect size was determined, known as partial eta squared (*η_p_*^2^), categorising the magnitude of the difference as trivial (*η_p_*^2^ ≤ 0.01), small (0.01 ≤ *η_p_*^2^ < 0.06), moderate (0.06 ≤ *η_p_*^2^ < 0.14), or large (*η_p_*^2^ ≥ 0.14) [23], as well as the statistical power (SP) of the data. In addition, linear regression and correlation models were employed to establish a relationship between the number of repetitions and the velocity loss percentage in each of the sets. To analyse muscle fatigue through velocity loss, a Student’s *t*-test for related samples was performed (Pre–Post BP exercise protocol).

Intrasubject variability between T2 and T3 was examined employing the standard error of measurement (SEM) using the equation SEM = SD √(1 − ICC), where ICC is the intra-class correlation coefficient. In addition, Bland Altman’s systematic bias ± random error and the coefficient of variation (CV), expressed as a percentage of the mean results, were used [24]. The range for classification of CV was less than 8.6% [25]. A *t*-test for related samples was performed to find differences between the test (T2) and retest (T3). The intra-class correlation coefficient (ICC) was also calculated at a 95% confidence interval (CI). ICC outcomes were classified as follows: excellent reliability (ICC ≥ 0.90), good reliability (0.90 > ICC ≥ 0.70), fair reliability (0.70 > ICC ≥ 0.40), and poor reliability (ICC < 0.40) [26]. The calculation was performed with α = 0.05 (5% chance of type I error) and 1 − β = 0.80 (80% power), applying the results of previous studies in which the sample size was the same or smaller. The calculated sample size was 25 subjects. All data are expressed as means, standard deviations (SD), 95% confidence intervals (CI), and minimum–maximum ranges (Min–Max). The level of significance was set to *p* < 0.05. All statistical tests were performed using SPSS v.25.0 (SPSS, Chicago, IL, USA).

## 3. Results

The 1RM obtained in the total group of participants was 79.73 ± 20.87 kg with 95% CI: 71.94–87.53 kg. The MPV 1RM was 0.18 ± 0.08 m·s^−1^ with 95% CI: 0.14–0.21 m·s^−1^. Table 1 shows the differences detected in the different variables between the sets of the exercise protocol. Significant differences were identified (*p* < 0.001) in all variables. After performing the post hoc test with Bonferroni’s adjustment, significant differences were found in all sets for 70% MPV Rep, MPV_rep_ Best and % loss MPV Set (*p* < 0.001), whereas in MPV_rep_ Last, there were only significant differences between set 1 and set 3 (*p* < 0.045).

**Figure 2:**
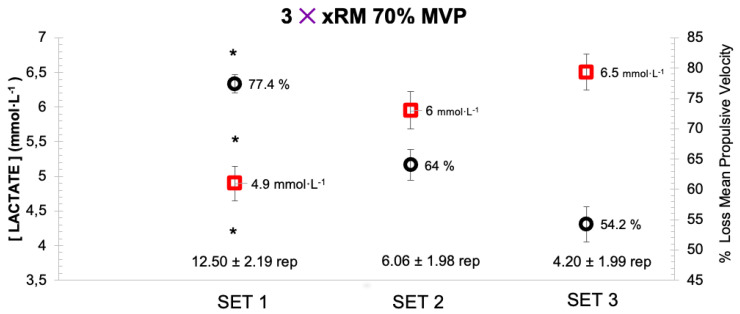
Comparison of the number of repetitions performed with velocity loss percentage and blood lactate level. * = significant difference between all sets (*p* < 0.05).

Relating the blood lactate concentrations to the velocity loss percentage of each set, it was observed that while the lactate levels increased significantly (*p* < 0.05), the velocity loss decreased significantly (*p* < 0.05). This is due to the fact that MPVrep Best decreases with the progressing sets; therefore, the velocity loss of the set also decreases. However, this increase in metabolic stress is in line with a significant decrease (*p* < 0.05) of the repetitions performed (Figure 2).

**Figure 3:**
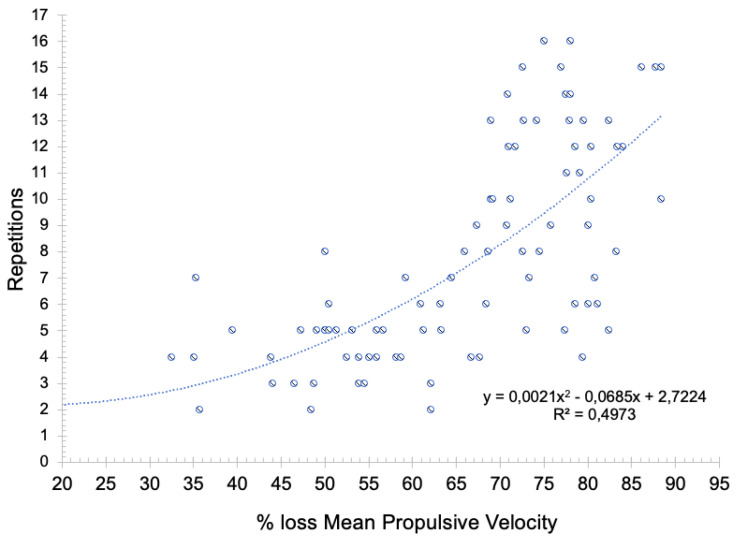
Linear regression between the number of repetitions performed and the velocity loss percentage of each set of the exercise protocol.

After conducting a regression analysis to establish an association between the number of repetitions and the loss of MPV performed in each set, it can be asserted that such a relationship is moderate (*R*^2^ = 0.497, *p* < 0.001) (Figure 3), with a strong correlation (*R* = 0.728, *p* < 0.001).

An analysis by strength level (Table 2) showed significant differences in all variables in the time factor (*p* < 0.05). After carrying out a pairwise comparison through Bonferroni’s post hoc adjustment, significant differences were obtained between all sets for 70% MPV Rep and MPV_rep_ Best (*p* < 0.001), whereas for the % loss MPV Set there were significant differences between set 1 and set 2, and between set 1 and set 3 (*p* < 0.001), and the difference between set 2 and set 3 was not significant (*p* = 0.051). Moreover, for MPV_rep_ Last there was only an approximation to statistical significance between set 1 and set 3 (*p* = 0.052). For the effect of the interaction, there were only significant differences in MPV_rep_ Best (*p* < 0.001). The pairwise comparison showed differences in the High RSR group in set 1 with set 2 and set 3 (*p* = 0.006, *p* < 0.001, respectively) and between set 2 and set 3 (*p* = 0.024). For Medium RSR and Low RSR, there were significant differences in set 1 with set 2 and set 3 (*p* < 0.001) and between set 2 and set 3 (*p* = 0.003). There was no statistical significance in any of the variables for the group factor. Moreover, regarding the interaction between the strength groups for each of the sets, there was statistical significance only between High RSR and Low RSR in set 1 (*p* = 0.008).

**Figure 4:**
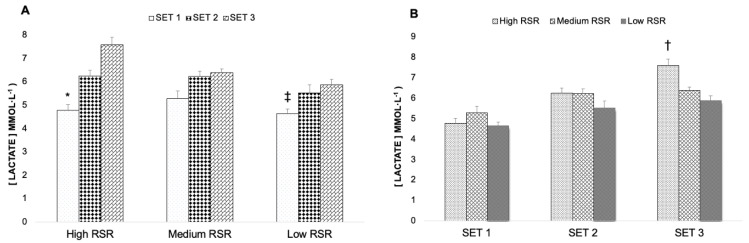
(**A**) Comparison of the blood lactate concentrations between strength level groups for each set, (**B**) comparison of blood lactate between sets for each strength level group. * = significant difference between all sets (*p* < 0.05). ‡ = significant difference between set 1 and set 3 (*p* < 0.05). † = significant difference between High RSR and Low RSR (*p* < 0.05).

Analysing the blood lactate concentrations between sets for each strength level group, significant differences were observed between sets (F (3,27) = 364.045, *p* < 0.001, *η_p_*^2^ = 0.931, SP = 1.000), but not for the group factor. However, analysing the effect of the set x group interaction, there was an approximation to statistical significance (F (6,27) = 2.192, *p* = 0.067, *η_p_*^2^ = 0.140, SP = 0.675). After performing Bonferroni’s post hoc test for pairwise comparison, it was observed that, for the highest strength level, there were significantly different lactate concentrations between all the sets (*p* < 0.05). Moreover, for Low RSR, there was statistical significance between set 1 and set 3 (*p* = 0.043) (Figure 4A). Moreover, comparing the lactate concentrations of each of the sets between strength groups, significant differences were only found between High RSR and Low RSR (*p* = 0.025) in set 3 (Figure 4B).

**Figure 5:**
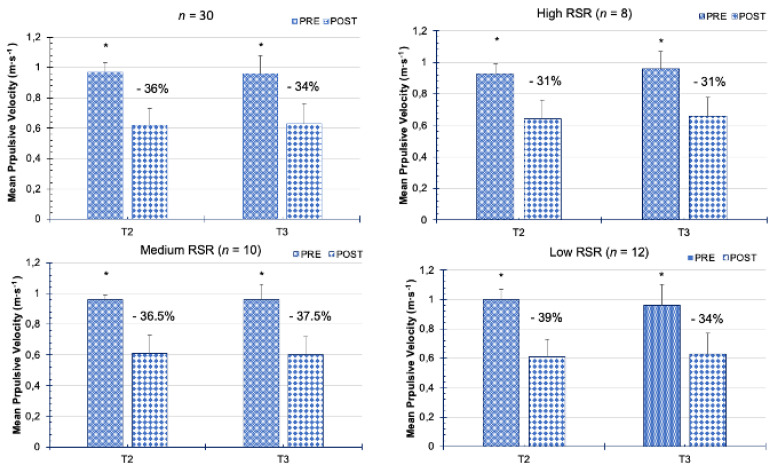
Mean Propulsive Velocity at 1 m·s^−1^ Tests pre and post bench press exercise protocol. T2 = second session TEST; T3 = third session RETEST. * = significant difference between pre and post exercise protocol (*p* < 0.05).

Figure 5 shows the muscle fatigue generated after performing the BP exercise protocol. The velocity loss in all participants was 36% in T2 and 34% in T3, with an acceptable intrasubject variability in the values of MPV at 1 m·s^−1^ (MPV at 1 m·s^−1^ pre-exercise: SEM ≤ 0.09 m·s^−1^, CV = 9.8%; MPV at 1 m·s^−1^ post-exercise: SEM ≤ 0.07 m·s^−1^, CV = 11.7%). After dividing the participants into the different strength levels, similar values were obtained in both velocity loss (31–39%) and intrasubject variability between the pre and post exercise of T2 and T3 (SEM ≤ 0.11 m·s^−1^, CV = 8–14.1%).

Table 3 presents the test–retest variability. In the analysis of the whole sample, significant differences were found in MPV_rep_ Best of set 2 and set 3 (*t* = −0.962, *p* = 0.006, SEM ≤ 0.06 m·s^−1^, CV = 13.3%, *t* = −2.161, *p* = 0.039, SEM ≤ 0.06 m·s^−1^, CV = 14.7%, respectively). For the High RSR group, there were significant differences only in the number of repetitions of set 2 (*t* = −3.055, *p* = 0.018, SEM ≤ 0.66 m·s^−1^, CV = 8.4%). For the Medium RSR group, there were significant differences between T2 and T3 in MPV_rep_ Best of set 2 and set 3 (*t* = −3.082, *p* = 0.013, SEM ≤ 0.06 m·s^−1^ CV = 13.5%, *t* = −3211, *p* = 0.011, SEM ≤ 0.05 m·s^−1^, CV = 12.7%, respectively). Lastly, for the Low RSR group, there were significant differences in MPV_rep_ Last of set 1 (*t* = −2.422, *p* = 0.034, SEM ≤ 0.05 m·s^−1^, CV = 28.3%). It was also observed that the intrasubject variability was similar between all groups in MPV_rep_ Best. However, for the number of repetitions and MPV_rep_ Last of set 1, the intrasubject variability decreased (lower SEM) with the increasing strength level (High RSR, SEM ≤ 0.81 repetitions, Medium RSR, SEM ≤ 2.07 repetitions, Low RSR, SEM ≤ 3.53 repetitions, High RSR, SEM ≤ 0.02 m·s^−1^, Medium RSR, SEM ≤ 0.04 m·s^−1^, Low RSR, SEM ≤ 0.04 m·s^−1^, respectively). Nevertheless, this variability changed in set 3, since it was lower in Low RSR (SEM ≤ 0.48 repetitions, SEM ≤ 0.02 m·s^−1^) and higher in High RSR (SEM ≤ 1.23 repetitions, SEM ≤ 0.08 m·s^−1^). The reliability of the variables measured at T2 and T3 for the total group of the participants was poor for the number of repetitions at set 1 (ICC = 0.299) and fair reliability for set 2 and set 3 (ICC = 0.777, ICC = 0.746, respectively). However, for the High RSR set 1 and 2 had good reliability (ICC = 0.850, ICC = 0.0894, respectively), while there was fair reliability for set 3 (ICC = 0.684). There was a fair reliability for the number of repetitions for all three runs of the Medium RSR (ICC = ~0.600). For the Low RSR, the number of repetitions of Set 2 and 3 had good reliability (Set 2, ICC = 0.741, Set 3, ICC = 0.881), while the number of repetitions of set 1 did not have good reliability (ICC = −0.561).

The CV was low or acceptable for MPV_rep_ Best both for the whole sample and in the division by strength level (CV = 8.2–15.9%). Acceptable values (CV = 18.9–21.9%) were also observed in the entire sample regarding the number of repetitions. However, in the division by strength groups, there were slightly higher CV values in set 1 for Low RSR (29%), and in set 2 and set 3 for Medium RSR (25.3% and 27%, respectively).

**Figure 6:**
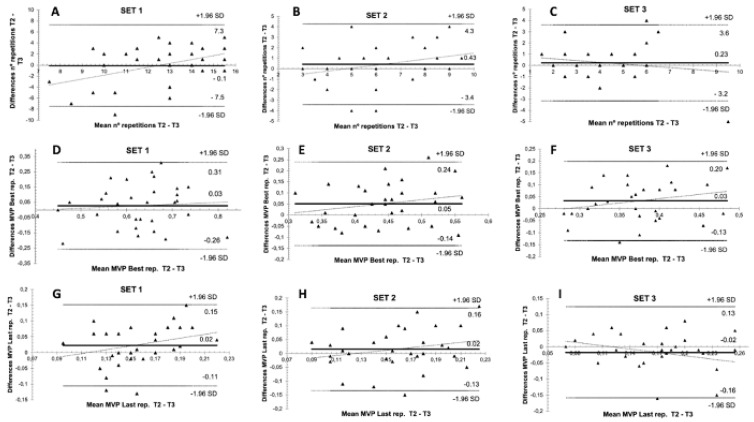
The Bland–Altman plots in the intrasubject variability [test (T2)–retest (T3)] assessment for the three sets of the bench press exercise protocol of the total group of participants (*n* = 30): (**A**–**C**) number of repetitions, (**D**–**F**) Mean propulsive velocity attained at the best repetition, (**G**–**I**) Mean propulsive velocity attained at the last repetition (MPV Last).

Using the Bland–Altman plots to express, in a different manner, the intrasubject variability in the test–retest, low systematic biases of the three analysed variables were obtained in all RSR groups (Table 4). These systematic biases were also low when the Bland–Altman plots were used in the whole sample (Figure 6).

## 4. Discussion

One of the main findings of this study was that, after implementing a strength protocol following the traditional training recommendations (3 sets at MNR with the same relative intensity 70% MPV at 1RM and a rest time of 2 min), the number of repetitions performed in each set was significantly different. That is, the MNR performed in the set for the total group of participants was 12.50 ± 2.19 repetitions in set 1, 6.06 ± 1.98 repetitions in set 2, and 4.20 ± 1.99 repetitions in set 3; thus, it was not possible to complete the same number of repetitions in each set for the same absolute load. This MNR also shows an important coefficient of variation in each of the sets and between RSR groups, which indicates that this variable can have an individual response depending on the capacity of each subject to recover from the preceding maximum effort. The control of these variables may be key to adequately analyse the conducted training and establish stronger relationships between the dose applied and its effects.

Moreover, it was detected that the MPV_rep_ Best of the first set was 0.62 ± 0.10 m·s^−1^, which is consistent with the values of previous studies at 70% 1RM [1,10,27], approximating the number of repetitions that are usually prescribed with such relative intensity [5,9,10]. With the repetitions performed at a MPV of ~0.62–0.64 m·s^−1^ (~12 repetitions, coinciding with 70% 1RM), the recommendations regarding the number of sets to be performed vary from 2 to 4, with rests of 2–3 min [5], or 1–3 sets with rests of 1–2 min in novice and intermediate training and 3–6 sets with rests of 2–3 min in advanced training [9].

The aim was to replicate the recommendations of such protocols, maintaining the same number of repetitions at 70% 1RM for the three sets. However, due to the decrease in the number of repetitions in set 2 and set 3, the volume for these sets was not as initially expected, since the number of repetitions was reduced to half in set 2, and to a third in set 3. This would be related to the MPV_rep_ Best, since it was significantly lower in set 2 (0.42 ± 0.07 m·s^−1^) and in set 3 (0.36 ± 0.06 m·s^−1^) with respect to set 1. This is a clear indicator of a reduction in the participants’ capacity to apply force as a result of the fatigue produced by the preceding load, with rests of 2 min being insufficient to maintain the strength values obtained with the same absolute load in the first set. Therefore, maintaining this same absolute load with two minutes of recovery, the degree of effort is different between each of the sets, which makes it difficult to quantify and control the desired training load with similar protocols.

Therefore, it was observed that the muscle fatigue generated in the first set did not allow repeating the number of repetitions in the second and third sets. Such fatigue is reflected in the fact that the MPV_rep_ Best is lower than 0.62 ± 0.10 m·s^−1^ at the beginning of set 2 and 3, which is not in line with the values of previous studies [27]. This also shows an important increase in the variation of the MNR with the successive sets (set 1, CV = 17.5%; set 2, = 32.7%; set 3, CV = 47.4%), which could indicate that this behaviour may depend on individual variables; this is possibly due to the fact that the muscle fatigue generated in each set and its relationship with the capacity to recover in the established time could be different in each participant. In the division by strength level, a similar increase in the variation of the MNR was observed. The verification of this important reduction in the capacity to apply force, linked to the generated fatigue, can also be observed in the MPV values of previous studies with the load of 1 m·s^−1^ [19] for such objective, which would represent approximately a mean relative intensity of about 45%, showing that the MPV obtained decreased by 31–39%. With respect to this increase in the fatigue level, a high metabolic stress level was reported in the entire group of participants, which increased with the successive sets. Furthermore, in the analysis by strength groups, the lactate concentrations in set 1 were significantly lower than in set 3 in High RSR and Low RSR. In addition, there was a significant difference in the lactate levels between High RSR and Low RSR in set 3. Therefore, this variation of the lactate levels between the sets and the different RSR groups could be another argument to justify the increase in the variation of MNR with the successive sets.

On its part, the MPV_rep_ Last dropped to 0.14 ± 0.04 m·s^−1^. This fact indicates that each participant performed his MNR in each set. The same occurred with the MPV_rep_ Last in set 2 and 3, where the MPV of 1RM for this exercise was reached (0.15 ± 0.05 m·s^−1^ and 0.18 ± 0.07 m·s^−1^, respectively). These values of MPV_rep_ Last are similar to those reported in previous studies [10,28].

Considering all of the above mentioned and the values of % loss MPV, there were also significantly different percentages between set 1 (~77.5% of MPV), set 2 (64%) and set 3 (~54%). The velocity loss percentage of set 1 is in line with the data of previous research [~79.2% ± 4.7 (70.5–90.1)] [27]. Moreover, 50% of the variation of the MPV loss can be explained by the number of repetitions, with a strong correlation between both variables (*R* > 0.7). That is, as the number of repetitions performed in the set decreased, the velocity loss was lower, since the MPV_rep_ Best in set 2 and 3 was also lower. Thus, in addition to the fact that it can be accurately used to monitor the training volume and being related to the percentage of repetitions performed with respect to the possible number of repetitions that can be completed, this variable could also be proposed as an indicator of intra-set fatigue, considering the reduction in the capacity to apply force throughout a number of sets and the possibility to control it through the velocity loss [27].

In view of these findings, the % loss MPV could be used as a better way of controlling training volume [28], and, depending on the velocity loss percentage reached, a different recovery time could be established in order to ensure that the MPV_rep_ Best of each of the programmed sets is similar. This would allow completing certain strength protocols with greater precision, as well as establishing more adequate dose–response relationships and much more rigorous recommendations. For instance, this would be the case of breast cancer survivors who suffer an important degree of fatigue due to the treatments they receive. Several studies have reported significant gains with protocols of three sets with 8–12 RM and 2 min of rest [29]. As has been documented, these protocols are not only difficult to reproduce rigorously, showing no better results in the improvement of strength [30,31], but they also involve an important fatigue, which should be controlled through a greater control of the applied stimulus and recoveries much better adjusted to the possibilities or individual needs of each subject. Further research is required to determine different recovery times as a function of the velocity loss.

With regard to the intrasubject variability, the variables showed different behaviours. For the number of repetitions in the three sets, the range was ~19–22%, whereas in the MPV_rep_ Best it was ~13–15%, with these CVs being considered acceptable. However, for the MPV_rep_ Last, these values were high (~26–38%). The values of set 1 were slightly higher than those reported in previous studies [10,32,33] and the range established for isoinertial studies [25], although the mentioned authors did not perform the measurements using optoelectronic devices. The SEM values were low, but they were also higher than those reported in the literature [10,33]. The SEM remained stable for the MPV_rep_ Best in each of the sets, whereas for the number of repetitions and for the MPV_rep_ Best of set 1, the SEM was very low in the High RSR group, and it increased with the decreasing strength level. On the other hand, the opposite was observed in set 3; that is, there was very little variation in Low RSR, and the increase in the SEM was towards the higher strength levels. The analysis of the intrasubject variability in T2 and T3 was completed with the Bland–Altman plots, obtaining results in line with the SEM and the CVs. This shows the consistency or stability of the observed measures, with the intrasubject variability, which could be due to an individual response of the fatigue generated in these tests, as that response could modify such values.

All this information allows the confirmation of some important aspects in research or in those propositions that could be developed with similar models, that is, with an MNR at a certain absolute load associated with a relative intensity value (%1RM). In the case of research, the independent variable should not be indicated as a value of maximum repetitions performed by all the individuals in all sets (e.g., 12 RM), but it should rather reflect the real value of the repetitions performed by each individual and expressed in terms of the mean MNR of the group, with the deviation and the minimum and maximum ranges in each set of repetitions. Similarly, the monitoring of the MPV, indicating the values that correspond to the first and last repetition and VL, as well as the use of the effort index [34], would be a necessary condition to guarantee accurate knowledge of the proposed stimulus and to establish a correct dose–response relationship.

The present study was performed using relative intensities and recovery times representative of the general recommendations conducted in a single exercise using a male population. However, no women participated in this study. Future studies should analyse whether there are more significant differences that could be associated with factors such as sex or age.

## 5. Conclusions

The results of the present study show that certain protocols that propose the execution of the MNR in a series of sets with a rest of 2 min are difficult of reproduce and apply. Likewise, it is demonstrated that this way of defining and controlling the exercise intensity is also inaccurate, since the number of repetitions performed in each set was significantly different. Moreover, the level of fatigue generated through each set and its relationship with the capacity to recover in the established time could be different in each individual, showing an important coefficient of variation in each of the sets. Therefore, it is necessary in future studies to define and reflect, with a greater degree of precision, on the training programmed and/or performed by each of the subjects or the group of subjects, both at the research level and regarding recommendations for the prescription of exercise in any population.

## Figures and Tables

**Table 1:** Descriptive data of the different variables in the bench press exercise protocol of the total group of participants (*n* = 30).

Variable		SET 1			SET 2				SET 3				*F*	*η_p_* ^2^	*p*
	M ± SD	Min–Max	95% CI	CV	M ± SD	Min–Max	95% CI	CV	M ± SD	Min–Max	95% CI	CV		*SP*	
70% MPV Rep (n°)	12.50 ± 2.19 *	8–16	11.68–13.32	17.5%	6.06 ± 1.98	2–10	5.33–6.81	32.7%	4.20 ± 1.99	2–12	3.46–4.94	47.4%	259.681	0.900 1.000	<0.001
MPV_rep_ Best (m·s^−1^)	0.62 ± 0.10 *	0.45–0.91	0.58–0.66	16.1%	0.42 ± 0.07	0.26–0.60	0.39–0.44	16.7%	0.36 ± 0.06	0.29–0.49	0.34–0.38	16.7%	139.553	0.828 1.000	<0.001
MPV_rep_ Last (m·s^−1^)	0.14 ± 0.04 ‡	0.07–0.22	0.13–0.17	28.6%	0.15 ± 0.05	0.07–0.24	0.13–0.17	33.3%	0.18 ± 0.07	0.07–0.34	0.15–0.20	38.9%	4.367	0.131 0.734	0.017
% loss MPV Set	77.42 ± 5.77 *	68.90–88.40	75.18–79.66	7.5%	64 ± 14.24	32.50–88.40	58.48–69.52	22.3%	54.21 ± 15.76	0–81.10	48.10–60.32	29.1%	23.773	0.468 1.000	<0.001

MPV = Mean Propulsive Velocity; Rep = repetitions; MPV_rep_ Best = Mean propulsive velocity attained at the best repetition; MPV_rep_ Last = Mean propulsive velocity attained at the last repetition; M = mean ± SD = standard deviation; CI = confidence intervals; Min–Max = lowest value–highest value; CV = Coefficient of variation; *η_p_*^2^ = partial eta-squared; SP = statistical power. * = significant difference between all sets (*p* < 0.05). ‡ = significant difference between set 1 and set 3 (*p* < 0.05).

**Table 2:** Data related to the number of repetitions, the highest and the last repetition of the bench press exercise protocol with a load of 70% MPV obtained in the 1RM test for each individual.

Variable	Level of Strength	SET 1 (M ± SD, Min–Max 95% CI, CV)	SET 2 (M ± SD, Min–Max 95% CI, CV)	SET 3 (M ± SD, Min–Max 95% CI, CV)	*p* Time *η_p_*^2^ *SP*	*p* Group *η_p_*^2^ *SP*	*p* Group × Time *η_p_*^2^ *SP*
70% MPV Rep (n°)	High RSR (*n* = 8)	12.63 ± 2 10–15 11.01–14.24 15.8%	7.38 ± 2 4–10 6.01–8.74 27.1%	5.5 ± 2.88 2–12 4.13–6.87 52.4%	<0.001 *	0.167	0.209
	Medium RSR (*n* = 10)	11.90 ± 2.33 9–16 10.46–13.34 19.6%	5.60 ± 2.01 2–9 4.38–6.82 35.9%	3.60 ± 1.35 2–5 2.38–4.82 37.5%	0.904	0.124	0.105
	Low RSR (*n* = 12)	12.92 ± 2.19 8–16 11.60–14.24 17%	5.58 ± 1.68 4–8 4.47–6.70 30.1%	3.83 ± 1.40 2–6 2.72–4.95 36.6%	1.000	0.362	0.386
MPV_rep_ Best (m·s^−1^)	High RSR (*n* = 8)	0.53 ± 0.08 0.45–0.71 0.47–0.60 15.1%	0.44 ± 0.07 0.35–0.60 0.38–0.49 15.9%	0.38 ± 0.06 0.30–0.49 0.34–0.43 15.8%	<0.001 *	0.499	<0.001 *
	Medium RSR (*n* = 10)	0.61 ± 0.10 0.46–0.75 0.55–0.67 16.4%	0.41 ± 0.06 0.29–0.49 0.36–0.45 14.6%	0.34 ± 0.04 0.29–0.42 0.30–0.38 11.8%	0.875	0.050	0.393
	Low RSR (*n* = 12)	0.68 ± 0.09 0.55–0.91 0.62–0.73 13.2%	0.41 ± 0.07 0.26–0.54 0.37–0.46 17.1%	0.35 ± 0.07 0.29–0.49 0.32–0.46 20%	1.000	0.158	0.998
MPV_rep_ Last (m·s^−1^)	High RSR (*n* = 8)	0.13 ± 0.04 0.07–0.19 0.10–0.16 30.8%	0.15 ± 0.06 0.07–0.24 0.11–0.18 40%	0.18 ± 0.09 0.07–0.34 0.13–0.23 50%	0.021 *	0.776	0.987
	Medium RSR (*n* = 10)	0.15 ± 0.03 0.09–0.18 0.12–0.17 20%	0.15 ± 0.04 0.09–0.21 0.12–0.18 26.7%	0.17 ± 0.07 0.07–0.23 0.13–0.22 41.2%	0.133	0.019	0.339
	Low RSR (*n* = 12)	0.15 ± 0.04 0.08– 0.22 0.12–0.17 26.7%	0.16 ± 0.05 0.07–0.24 0.13–0.19 31.3%	0.18 ± 0.05 0.08–0.23 0.14–0.22 27.8%	0.709	0.086	0.066
% loss MPV Set	High RSR (*n* = 8)	75.38 ± 7.13 68.90–87.72 71.24–79.51 9.5%	61.63 ± 20.07 32.50–88.40 50.92–72.34 32.57%	52.83 ± 24.92 0–81.10 40.93–64.74 47.17%	<0.001 *	0.646	0.996
	Medium RSR (*n* = 10)	76.54 ± 6.46 69.2–80.40 72.65–80.44 8.4%	64.44 ± 12.51 49.10–78.57 54.34–74.54 19.4%	54.73 ± 12.17 39.50–77.40 43.50–65.95 22.2%	0.462	0.034	0.003
	Low RSR (*n* = 12)	79.63 ± 5.67 70.90–88.40 76.10–83.16 7.1%	65.36 ± 14 35.10–80.77 56.23–74.50 21.4%	54.79 ± 10.72 35.70–80.10 44.64–64.95 19.6%	1.000	0.114	0.058

MPV = Mean Propulsive Velocity; RSR = Relative Strength Ratio, defined as 1RM value divided by body mass; Rep = repetitions; MPV_rep_ Best = Mean propulsive velocity attained at the best repetition; MPV_rep_ Last = Mean propulsive velocity attained at the last repetition; M = mean ± SD = standard deviation; CI = confidence intervals; Min–Max = lowest value–highest value; CV = Coefficient of variation. *η_p_*^2^ = partial eta-squared; SP = statistical power. * = significant difference (*p* < 0.05).

**Table 3:** Intrasubject variability in the number of repetitions of the bench press exercise protocol performed, best repetition and last repetition, on two different days for the four strength levels.

	Repetitions SET 1 (n°)				Repetitions SET 2 (n°)				Repetitions SET 3 (n°)			
	T2	T3	SEM	CV	T2	T3	SEM	CV	T2	T3	SEM	CV
All RSR (*n* = 30)	12.50 ± 2.19	12.40 ± 3.42	2.35	18.9%	6.07 ± 1.98	6.50 ± 2.52	1.06	16.9%	4.20 ± 1.99	4.43 ± 1.76	0.95	21.9%
High RSR (*n* = 8)	12.63 ± 2.00	13.63 ± 2.2	0.81	6.2%	7.38 ± 2 *	8.38 ± 2.07	0.66	8.4%	5.50 ± 2.9	5.75 ± 1.49	1.23	21.9%
Medium RSR (*n* = 10)	11.90 ± 2.33	12.60 ± 4.17	2.07	16.9%	5.60 ± 2.01	6.40 ± 2.68	1.52	25.3%	3.60 ± 1.35	4.50 ± 1.90	1.10	27%
Low RSR (*n* = 12)	12.92 ± 2.28	11.42 ± 3.37	3.53	29%	5.58 ± 1.68	5.33 ± 2.02	0.94	17.3%	3.82 ± 1.47	3.55 ± 1.29	0.48	12.9%
	**MPV_rep_ Best SET 1 (m·s^−1^)**				**MPV_rep_ Best SET 2 (m·s^−1^)**				**MPV_rep_ Best SET 3 (m·s^−1^)**			
	**T2**	**T3**	**SEM**	**CV**	**T2**	**T3**	**SEM**	**CV**	**T2**	**T3**	**SEM**	**CV**
All RSR (*n* = 30)	0.62 ± 0.11	0.64 ± 0.12	0.1	15.2%	0.42 ± 0.07 *	0.47 ± 0.09	0.06	13.3%	0.36 ± 0.06 *	0.39 ± 0.08	0.06	14.7%
High RSR (*n* = 8)	0.53 ± 0.08	0.60 ± 0.11	0.09	15.2%	0.44 ± 0.07	0.46 ± 0.07	0.04	8.3%	0.38 ± 0.06	0.41 ± 0.07	0.04	11.1%
Medium RSR (*n* = 10)	0.61 ± 0.10	0.68 ± 0.11	0.1	15%	0.41 ± 0.06 *	0.49 ± 0.08	0.06	13.5%	0.34 ± 0.04 *	0.41 ± 0.05	0.05	12.7%
Low RSR (*n* = 12)	0.68 ± 0.10	0.64 ± 0.13	0.1	14.1%	0.41 ± 0.08	0.45 ± 0.11	0.07	15.2%	0.35 ± 0.07	0.36 ± 0.09	0.06	15.9%
	**MPV_rep_ Last SET 1 (m·s^−1^)**				**MPV_rep_ Last SET 2 (m·s^−1^)**				**MPV_rep_ Last SET 3 (m·s^−1^)**			
	**T2**	**T3**	**SEM**	**CV**	**T2**	**T3**	**SEM**	**CV**	**T2**	**T3**	**SEM**	**CV**
All RSR (*n* = 30)	0.14 ± 0.04	0.16 ± 0.05	0.05	31%	0.15 ± 0.06	0.17 ± 0.06	0.06	37.7%	0.18 ± 0.07	0.16 ± 0.05	0.04	25.8%
High RSR (*n* = 8)	0.13 ± 0.04	0.13 ± 0.03	0.02	13.4%	0.15 ± 0.06	0.14 ± 0.04	0.06	40.3%	0.18 ± 0.09	0.13 ± 0.05	0.08	52.3%
Medium RSR (*n* = 10)	0.15 ± 0.03	0.16 ± 0.03	0.04	22.4%	0.15 ± 0.04	0.17 ± 0.06	0.04	25.8%	0.17 ± 0.07	0.16 ± 0.05	0.04	23.7%
Low RSR (*n* = 12)	0.15 ± 0.04 *	0.20 ± 0.06	0.05	28.3%	0.16 ± 0.05	0.18 ± 0.07	0.07	39.5%	0.18 ± 0.05	0.18 ± 0.06	0.02	11.5%

MPV = Mean Propulsive Velocity; RSR = Relative Strength Ratio, defined as 1RM value divided by body mass; Rep = repetitions; MPV_rep_ Best = Mean propulsive velocity attained at the best repetition; MPV_rep_ Last = Mean propulsive velocity attained at the last repetition. Data expressed as means ± standard deviation. SEM = Standard Error of Measurement; CV = Coefficient of variation. * = significant difference (*p* < 0.05).

**Table 4:** The Bland–Altman plots in the intrasubject variability [test (T2)–retest (T3)] assessment of the bench press exercise protocol performed, best repetition and last repetition on two different days for the four strength levels.

	Repetitions SET 1 (n°)			Repetitions SET 2 (n°)			Repetitions SET 3 (n°)		
	Systematic Bias	Random Error	CI (95%)	Systematic Bias	Random Error	CI (95%)	Systematic Bias	Random Error	CI (95%)
High RSR (*n* = 8)	1	1.31	3.62 to −1.62	1	0.93	2.85 to −0.85	0.25	2.31	4.88 to −4.38
Medium RSR (*n* = 10)	0.7	3.68	8.07 to −6.67	0.8	2.57	5.95 to −4.35	0.9	1.79	4.48 to −2.68
Low RSR (*n* = 12)	−0.5	4.50	7.51 to −10.51	−0.25	1.71	3.18 to −3.68	0	1.28	2.56 to −2.56
	**MPV_rep_ Best SET 1 (m·s^−1^)**			**MPV_rep_ Best SET 2 (m·s^−1^)**			**MPV_rep_ Best SET 3 (m·s^−1^)**		
	**Systematic Bias**	**Random Error**	**CI (95%)**	**Systematic Bias**	**Random Error**	**CI (95%)**	**Systematic Bias**	**Random Error**	**CI (95%)**
High RSR (*n* = 8)	0.07	0.13	0.33 to −0.19	0.02	0.07	0.16 to −0.12	0.03	0.07	0.17 to −0.11
Medium RSR (*n* = 10)	0.07	0.13	0.34 to −0.20	0.09	0.09	0.27 to −0.09	0.07	0.07	0.20 to −0.07
Low RSR (*n* = 12)	−0.04	0.14	0.24 to −0.32	0.04	0.11	0.26 to −0.18	0.006	0.10	0.20 to −0.19
	**MPV_rep_ Last SET 1 (m·s^−1^)**			**MPV_rep_ Last SET 2 (m·s^−1^)**			**MPV_rep_ Last SET 3 (m·s^−1^)**		
	**Systematic Bias**	**Random Error**	**CI (95%)**	**Systematic Bias**	**Random Error**	**CI (95%)**	**Systematic Bias**	**Random Error**	**CI (95%)**
High RSR (*n* = 8)	−0.001	0.07	0.13 to −0.14	−0.008	0.08	0.15 to −0.16	−0.05	0.11	0.17 to −0.26
Medium RSR (*n* = 10)	0.01	0.05	0.11 to −0.09	0.02	0.06	0.14 to −0.10	−0.01	0.07	0.12 to −0.14
Low RSR (*n* = 12)	0.05	0.07	0.19 to −0.09	0.03	0.09	0.20 to −0.14	0.003	0.04	0.08 to −0.08

MPV = Mean Propulsive Velocity; RSR = Relative Strength Ratio, defined as 1RM value divided by body mass; Rep = repetitions; SEM = Standard Error of Measurement; MPV_rep_ Best = Mean propulsive velocity attained at the best repetition; MPV_rep_ Last = Mean propulsive velocity attained at the last repetition. Data expressed as means ± standard deviation. CV = Coefficient of variation.

## Data Availability

Not applicable.
